# Strength in arms: empowering older adults against the risk of slipping and falling—a theoretical perspective

**DOI:** 10.3389/fspor.2024.1371730

**Published:** 2024-03-08

**Authors:** Jonathan Lee-Confer

**Affiliations:** Department of Physical Therapy, University of Arizona, Tucson, AZ, United States

**Keywords:** aging, falls, physical exercise, balance, physical therapy, slips balance, slips, training

## Abstract

**Background:**

Slips and falls are a serious health concern, particularly among older adults. Current physical therapy protocols strengthen the legs to improve balance. However, arm movements help maintain balance during a slip incident. Understanding how arm movements improve balance may help clinicians develop more comprehensive fall-prevention protocols to improve patient outcomes.

**Clinical question:**

What limitations exist in current fall prevention protocols for reducing falls in older adults during slip incidents, and what new strategies can enhance these outcomes?

**Key results:**

Slip incidents often result in a sideways loss of balance, leading to hip fractures in older adults. During a slip, the legs do not produce sideways motion and are less effective in regaining balance in this direction. Contrary, the arms produce 100 + degrees of abduction and this motion reduces falls by 200%+ during a slip incident. Notably, older adults exhibit 35.7% decreased arm abduction acceleration responses compared to younger adults during a slip incident. This delay may be attributed to age-related decreases in type II fibers of the deltoid. High-velocity and ballistic training have been shown to improve the proportion and size of type II fibers as well as improve fall outcomes when focused on the lower extremities.

**Clinical application:**

Therefore, I propose incorporating arm abductor training, alongside leg exercises, as a cost-effective and low-risk intervention to enhance the slip responses in older adults. In light of its minimal risk and considerable potential benefits, starting arm abductor exercises with older adults is a sensible move.

## Introduction

Slips and falls are a serious health concern across the globe. It is reported that approximately 56% of older adults' falls result from a slip incident, which then leads to injuries and a lower quality of life (1). Furthermore, people spend more of their personal income on fall-related injuries than all cancers combined in the United States of America (2). Current physical therapy practices target strengthening of the legs to improve balance outcomes in older adults (3, 4). However, there has been an emerging body of literature that suggests the arms play a significant role in maintaining balance during a slip incident (5). As such, it is important to understand the utility of the arms in regaining balance from a slip incident and how the arms can potentially be trained to supplement the current physical therapy fall-prevention protocols. The purpose of this perspective is to review whole-body human movements during a slip incident, discuss the current fall-prevention protocols, and propose the arms' role in slip incidents to provide another perspective that can enhance our current fall-prevention paradigms to reduce the risk of adults falling from a slip incident. Understanding the mechanisms individuals use to recover from a slip may provide insight into developing low-cost interventions that can reduce significant injuries from occurring.

### Clinical question

What is a limitation of current physical therapy interventions for older adults to prevent falls during slip incidents, and what new strategies can improve these outcomes?

#### How the body moves during a slip incident

**Figure 1:**
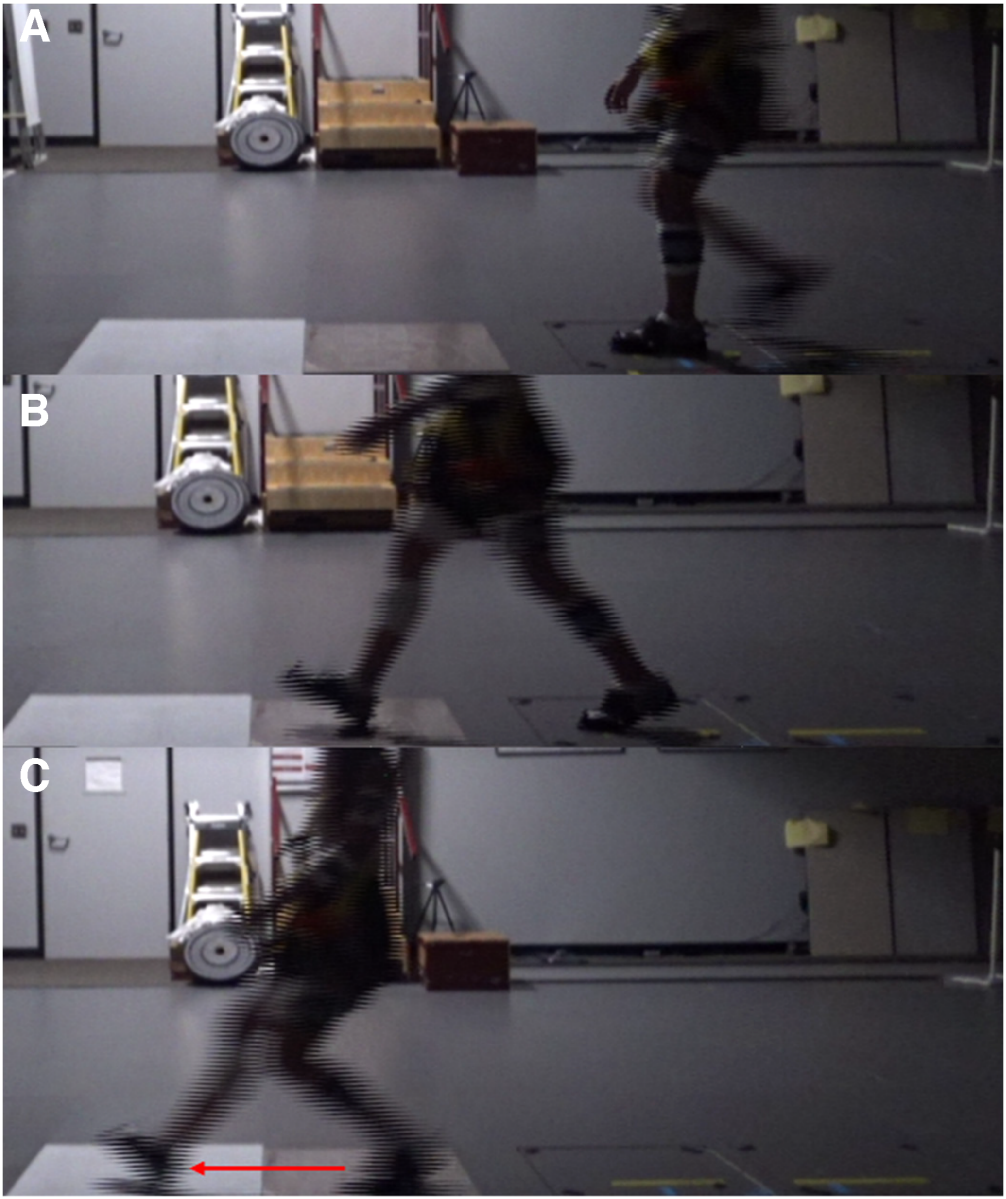
A series of photographs showing the chronological events of walking and leading up to, and after, a slip incident. (**A**) depicts the time point when the left foot is planted onto the ground and the right foot begins swing phase before landing on the contaminated tile. (**B**) exhibits the moment of heel strike onto the contaminated tile. (**C**) shows the maximal forwards distance the foot slides ahead of the body after a slip incident. The red arrow directed left indicates the direction and magnitude of the slipped foot.

**Figure 2:**
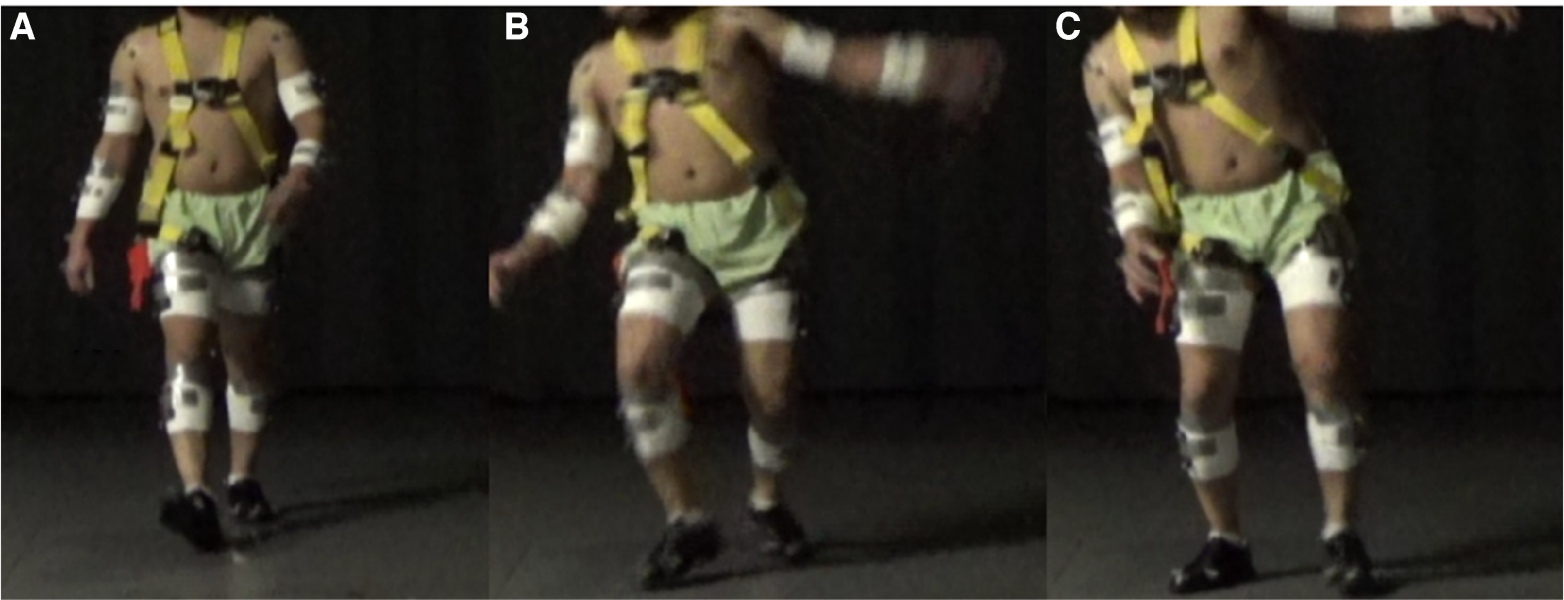
A frontal view time-series photograph of an individual experiencing a slip incident. (**A**) An individual steps onto a slippery surface with their right foot. (**B**) An individual's right foot slipped, and the body begins to rotate towards the slipped foot while the contralateral arm begins to raise. (**C**) An individual's body is sideways rotated towards the side of the slipped foot, and the individual's contralateral arm is abducted.

Individuals experience a sideways-directed loss of balance in response to a slip incident (6–9). During a slip, an individual is walking when the forward foot accepting weight steps onto a contaminant such as soapy water, cooking oil, or some other substance that lowers the friction of the floor. The anterior foot slides ahead of the individual (Figure 1) (10–13) and their body rotates to the side, towards the side of the slipped foot (Figure 2). The leg that experienced a slip must retract and pull itself directly back under the body since the feet serve as the base of support for the body (12, 14, 15). Individuals will experience a loss of balance if their center of mass leaves the base of support either from both feet ending up in front of the body (13), or if the center of mass moves too much to the side where the legs cannot support the body. A sideways loss of balance in older adults is considered potentially more hazardous, as a sideways loss of balance is known to lead to hip fractures on the greater trochanter of the femur (16–19). Because of that, finding ways to minimize a sideways loss of balance is the primary key to reducing hip fractures. As the legs serve as the base of support for the body during a slip, the following section will discuss the research on training the legs and the efficacy on fall prevention.

#### The lower extremities' role in physical therapy and slip incidents

The legs have received a substantial amount of attention in physical therapy research when it comes to balance and its applications in rehabilitation. It is intuitive that researchers dedicate their efforts to focus on the legs as studies report that increases in leg strength have direct improvements in maintaining balance. In fact, dozens of studies report interventions that highlight the clear benefits of leg strengthening exercises, standing balance tasks, and Time Up and Go (TUG)-type walking tests on fall prevention in older adults, making leg strengthening a cornerstone for physical therapy fall prevention protocols (3, 4, 20–24). More specifically, many of these interventions focus on resistance training for knee flexors, hip abductors, ankle dorsi/plantar flexors, and knee extensors. Functionally, many of these programs emphasize TUG, walking sideways, walking backwards, and sit-to-stand exercises. Inherently, improving stability and strength of the lower extremities is paramount to promote improved movements to regain balance from a slip incident by allowing older adults to generate rapid and high forces of their leg muscles to pull the slipped leg back towards the body. However, the legs' role in controlling a sideways loss of balance is questionable as a slip makes the body rotates within the frontal plane, and a sideways loss of balance requires sideways movements to maintain balance. The legs do not produce sideways motion during a slip incident (10, 13, 25, 26) and are unlikely to contribute to the restoration of balance from a slip in the sideways direction for two reasons: (1) large abduction movements of one leg would reduce the base of support to one foot and increase the likelihood of falling, and (2) the hip range of motion is about 37 degrees (27) which may not provide sufficient motion to counter the lateral rotation of the body. As mentioned previously, maintaining stability from a sideways loss of balance is critical to prevent hip fractures as hip fractures are known to occur from a sideways loss of balance. As such, the arms are the likely segments that will aid in regaining balance from a sideways-directed loss of balance as the arms can produce 160–180 degrees of abduction (28, 29) with individuals exhibiting approximately 61.7 ± 26.9 degrees of abduction during a slip incident. Furthermore, the arms account for approximately 10% of the body's mass (30) making them a strong contender for facilitating quick and rapid movements to adjust to the center of mass position and maintaining balance within the frontal plane.

#### The role of the arms in a slip incident

The arms are critical for maintaining balance during a slip incident. The earliest research reporting abduction of the arms during an overground slip incident was published in 2022 (5). Through more recent work, we know that this arm abduction reduces the lateral excursion of the center of mass by 37.5% (31). This explains why slip incidents in young and healthy adults reduce their fall rate by 200%–300% when their arms are free to move compared to when they are constrained (32). The mechanical benefits of arm movements during a slip have significant effects on reducing sideways loss of balances in several biomechanical measurements of stability. However, the mechanical benefits of arm movements are not exhibited in older adults experiencing an overground slip incident. A study reported that older adults' center of mass moved 2.26× farther away from the initial position compared to young adults experiencing a slip incident even though they exhibited similar amounts of arm abduction (33). A key finding was that the older adults' arm acceleration was significantly slower and their time to peak arm movements was delayed by 310 ms compared to younger adults leading to a higher likelihood of a sideways loss of balance from a slip incident. A separate study also reported that older adults exhibited delayed reactive arm responses compared to younger adults during a slip incident (34). Currently, we know how the arms should move to restore balance after a slip incident, but it is currently unclear if the arms can be trained and how they should be trained.

#### Arm abductors in aging

The abductors of the arm may need to be an additional focus of resistance training in older adults for fall prevention protocols. Previous work reported a rapid observation of arm abduction in younger adults and slower arm abduction acceleration in older adults (5). The proportion of type IIb muscle fibers in the deltoid muscle was 2.58 times higher in males aged 50–59 compared to males aged 70–79 (35). The type IIb muscle fiber diameter was 1.58 times higher in females aged 50–59 compared to females aged 70–79 (35). Furthermore, this general effect of aging on decreases in type II fibers of the deltoid were also shown in a systematic review (36). The older adults' slower arm abduction responses could be due to a reduction size and decline in type IIb fibers within the deltoid muscle. It is reported that performing high-velocity ballistic movements for training may increase type II fibers rather than the convention exercises of lifting a weight at slow and controlled speeds (37). Therefore, it is possible that power training the abductors of the arm through rapid and ballistic movements may improve the capability to generate rapid arm abduction forces, reduce the reaction times to create abduction, and thus improve the ability to restore a sideways loss of balance from a slip incident. Older adults' restoration of balance could be improved significantly if they are trained appropriately to facilitate rapid arm responses during a slip perturbation.

#### The ability to train the arms in older adults

There are indirect measurements that suggest training older adults' arms to assist in resisting falls is a plausible strategy. One study demonstrated that older adults' reaction times to visual cues can improve with training (38), implying that the neural inputs and control of movements of older adults can adapt to proper stimuli. Furthermore, it is reported that the arm movements observed during a slip incident were recruited as early as 57 ms after slip initiation and may partially be initiated from active control (5), suggesting that the nervous system is actively initiating arm responses to a slip incident, and the arm responses have the potential to be trained. Lastly, perturbation training has been shown to be an effective tool against fall incidents (39–44). The idea that the nervous system can adapt to large perturbations also provides credence that the nervous system can be trained to produce more effective movements. As such, perturbation training likely requires a certain amount of lower extremity strength to be effective, and similarly, perturbation training could potentially be enhanced with strength improvements to the arm abductors. Furthermore, strength training in older adults above the age of 85 years old showed the ability to significantly improve muscle mass and strength meaning that improved adaptations are possible across the age range (45). Lastly, stroke survivors improved their arm speed and range of motion with rapid movement training (46). This suggests that adaptations are possible at considerably older ages and training the arms may be feasible for the older population.

This perspective should be read with caution as there are limitations to this opinion. While the lower extremities have received numerous experimental studies, meta-analyses and systematic reviews exhibiting the positive effects on balance from strength training, it is imperative to note that there are no intervention studies demonstrating the efficacy of arm abductor training and improved fall outcomes during a slip incident. This perspective to train the arm abductors through high-velocity and ballistic training is supported with indirect evidence, and the notion that arm abductor training may have positive effects on restoration of balance from a slip incident is theoretical at this juncture. However, I posit that there is clear evidence that the arms reduce a sideways loss of balance during a slip and adding a few minutes of strength training of the arm abductors is a low-cost and low-risk intervention for a possible avoidant of highly debilitating injuries. So, I ask the question, “Why not start training the arm abductors in older adults now?”

### Key points

#### Findings

Older adults exhibit significantly slower arm abduction responses compared to younger adults during slip incidents, potentially increasing their risk of side falls and severe injuries. Studies reveal a notable decline in type II muscle fibers in the deltoids of older adults, which can be countered through high-velocity and ballistic training.

#### Implications

This research challenges the current focus of fall prevention physical therapy, which primarily emphasizes leg strengthening, balance, and gait training, and overlooks the potential of arm training. Integrating low-risk, cost-effective arm abductor training into existing protocols could notably enhance fall recovery for older adults during slip incidents.

#### Caution

It is important to acknowledge the absence of direct intervention studies linking arm abductor training to improved fall outcomes during slip incidents. The advocated perspective is based on indirect evidence, necessitating further research for conclusive support.

## Data Availability

The original contributions presented in the study are included in the article/Supplementary Material, further inquiries can be directed to the corresponding author.
